# Clinical and Radiographic Findings and Usefulness of Computed Tomographic Assessment in Two Children with Regional Odontodysplasia

**DOI:** 10.1155/2014/764393

**Published:** 2014-08-27

**Authors:** Junko Matsuyama, Ray Tanaka, Futabako Iizawa, Tomiko Sano, Shoko Kinoshita-Kawano, Sachiko Hayashi-Sakai, Tomoe Mitomi

**Affiliations:** ^1^Division of Pediatric Dentistry, Graduate School of Medical and Dental Sciences, Niigata University, 2-5274 Gakkocho-dori, Chuo-ku, Niigata 951-8514, Japan; ^2^Division of Oral and Maxillofacial Radiology, Graduate School of Medical and Dental Sciences, Niigata University, 2-5274 Gakkocho-dori, Chuo-ku, Niigata 951-8514, Japan; ^3^Takarazuka Sanda Hospital, 2-22-10 Nishiyama, Sanda, Hyogo, 669-1537, Japan

## Abstract

Regional odontodysplasia is a rare, severe, and nonhereditary developmental disorder in tooth formation and involves epithelial and mesenchymal-derived dental tissue. On radiographs, affected teeth have an abnormal morphology, a hypoplastic crown, and only a faint outline of hard tissue, a condition termed “ghost teeth.” We report clinical and radiographic findings from two children with regional odontodysplasia. Using computed tomography (CT), we calculated attenuation coefficients (i.e., Hounsfield units) for affected teeth and assessed the condition of dental follicles. To measure density, regions of interest were delimited and CT values were calculated. In our two patients, the CT values for enamel were lower in affected teeth than in sound teeth, while CT values for dentin were similar for affected and sound teeth. The average CT value for dental follicles in affected teeth was about 65 to 120, which suggests that dense fibrous connective tissues or hard tissue-like structures might be present in dental follicles. Analysis of CT values may be quite useful in the diagnosis and treatment of regional odontodysplasia.

## 1. Introduction

Regional odontodysplasia (RO) was initially described by Hitchin in 1934 [1], and the term odontodysplasia was first proposed in 1963 [2]. RO is a rare but severe nonhereditary developmental disorder of tooth formation that involves epithelial and mesenchymal-derived dental tissue. It affects primary and permanent dentition and is localized unilaterally in one dental arch. Although the cause of RO is unknown, several hypotheses have been suggested, including trauma, nutritional deficiency, infection, metabolic abnormalities, systemic disease, and genetic effects [3]. Clinically, RO commonly accompanies delayed, failed, or partial tooth eruption, with or without gingival abscess. The enamel and dentin of affected teeth are hypoplastic and hypocalcified. Irregular pits and grooves are usually present on the surface of affected teeth, which are yellowish or brownish.

Radiographic images of affected teeth show an abnormal morphology, a hypoplastic crown, and only a faint outline of hard tissue, a condition referred to as “ghost teeth” [2]. There is reduced contrast between enamel and dentin, both of which have low radiodensity. The enamel and dentin layer are very thin, and the pulp chambers are abnormally large, with open apices and enlarged follicles. In some cases, unerupted teeth are surrounded by X-ray-permeable regions with clear boundaries, which resemble a cyst or tumor. Thinning of neighboring alveolar bone and decrease in trabeculae are also sometimes observed [4–6]. Although radiographic findings are important in diagnosing RO, few studies have reported findings from computed tomography (CT) imaging of RO [7, 8]. On CT images, attenuation coefficients, called Hounsfield units (HU), represent the extent of radiation absorption by structures, which is related to density [9]. Three-dimensional imaging and data from CT could greatly improve RO diagnosis and treatment. In this study, we report clinical and radiographic findings from two children with regional odontodysplasia. We used CT images to create depth profiles and calculate the attenuation coefficients of affected teeth and the condition of dental follicles.

## 2. Case Presentations

### 2.1. Case 1

**Figure 1:**
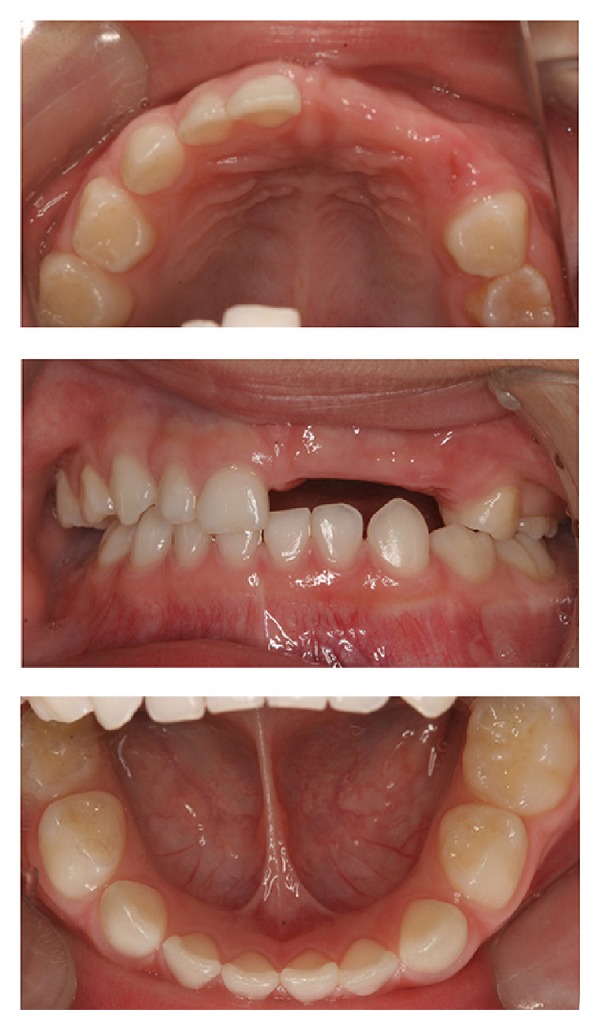
Intraoral findings at initial visit (case 1). An edentulous area is present in the area of the left primary maxillary central incisor, primary maxillary lateral incisor, and primary canine.

**Figure 2:**
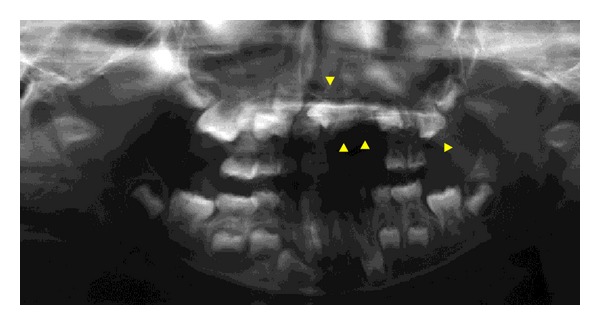
Panoramic radiograph at initial visit (case 1). Several permanent maxillary teeth are malformed and hypocalcified (arrowheads).

A Japanese boy aged 5 years and 8 months was referred to the Pediatric Dentistry Department of Niigata University Medical and Dental Hospital by a dentist at another clinic. The chief complaint was malformation of the anterior primary and permanent teeth. Extraoral examination revealed no significant abnormalities. His medical history was unremarkable, and no dental or maxillofacial abnormalities were reported in his family. His mother was well and had taken no medications during pregnancy. The patient presented with primary dentition. An edentulous area was observed in the left maxilla: the primary central incisor, primary lateral incisor, and primary canine were missing (Figure 1). These teeth had been extracted at his previous dental clinic as treatment for severe apical periodontitis. The primary maxillary left second molar was yellowish and had a slightly irregular surface. The other primary teeth appeared normal. A panoramic radiograph at the first examination showed that several permanent maxillary teeth (i.e., the central incisors, left lateral incisor, left canine, and left second premolar) were affected (Figure 2). These teeth were malformed and hypocalcified, had unusual morphology, and were small and hypoplastic. Furthermore, root formation was immature, and the roots were short. Dental follicles in the crowns of the permanent maxillary left central and lateral incisors were enlarged. The affected edentulous region was temporarily rehabilitated with a removable space maintainer, and the patient was periodically examined for permanent tooth formation.

**Figure 3:**
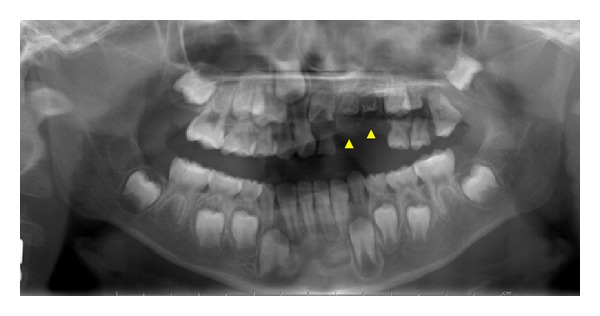
Panoramic radiograph at age 8 years and 4 months (case 1). Dental follicles of permanent affected teeth have irregularly expanded (arrowheads).

At age 8 years and 4 months, the permanent maxillary central incisors and left lateral incisor had not erupted, although other central and lateral incisors had erupted in the oral cavity. A part of the tooth crown of the permanent maxillary left first molar had erupted and was hypoplastic. Affected teeth had a diminished tendency to erupt, and a panoramic radiograph showed that the dental follicles of affected permanent teeth had expanded irregularly (Figure 3).

### 2.2. Case 2

**Figure 4:**
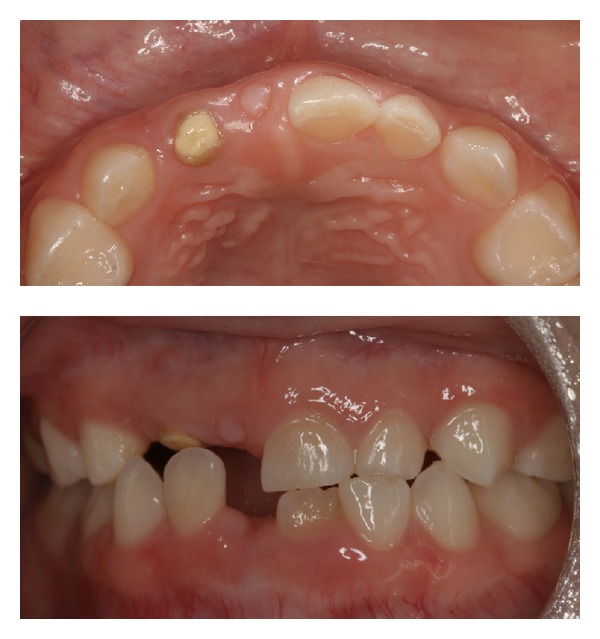
Intraoral findings at initial visit (case 2). The primary maxillary right central and lateral incisors are severely hypoplastic.

A Japanese boy aged 6 years and 1 month was referred to the Pediatric Dentistry Department of Niigata University Medical and Dental Hospital by his previous dentist. The chief complaint was malformation of the maxillary right primary and permanent central and lateral incisors. His medical history was unremarkable, and no dental or maxillofacial abnormalities were reported in his family. His mother was well and had taken no medications during pregnancy. Extraoral examination revealed no significant abnormalities. The patient presented with primary dentition. The primary maxillary right central and lateral incisors were severely hypoplastic, and only the tooth roots remained in the arch. The primary maxillary right lateral incisors were filled with composite or cement (Figure 4).

**Figure 5:**
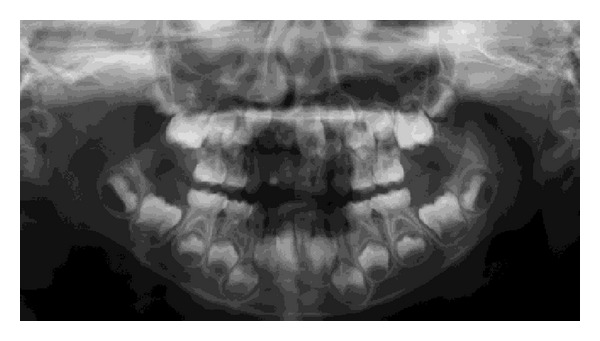
Panoramic radiograph at first visit (case 2). The permanent maxillary right central and lateral incisors are malformed and hypocalcified.

**Figure 6:**
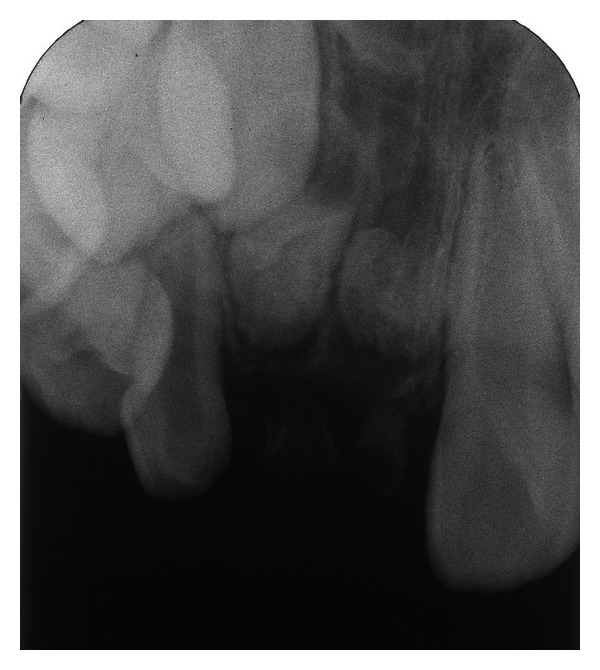
Periapical radiograph at age 9 years and 2 months (case 2). There is no evidence of eruption tendency.

A radiograph obtained at the first examination showed severe hypoplasia of the primary maxillary right central and lateral incisors. The permanent maxillary right central and lateral incisors were malformed and hypocalcified, had unusual morphology, and were small and hypoplastic. The dental follicles of the crowns of affected permanent teeth were enlarged (Figure 5). At age 8 years and 2 months, the permanent maxillary left central incisor had erupted, but the primary maxillary right central and lateral incisors remained; thus, the primary maxillary right central and lateral incisors were extracted. At age 9 years and 2 months, the permanent maxillary left lateral incisor had not erupted, although the contralateral incisor had erupted. A periapical radiograph obtained at the same age showed that the degree of calcification of the dental germs of affected incisors had not substantially changed since the initial diagnosis. In addition, there was no evidence of eruption tendency (Figure 6).

### 2.3. CT Findings

**Figure 7:**
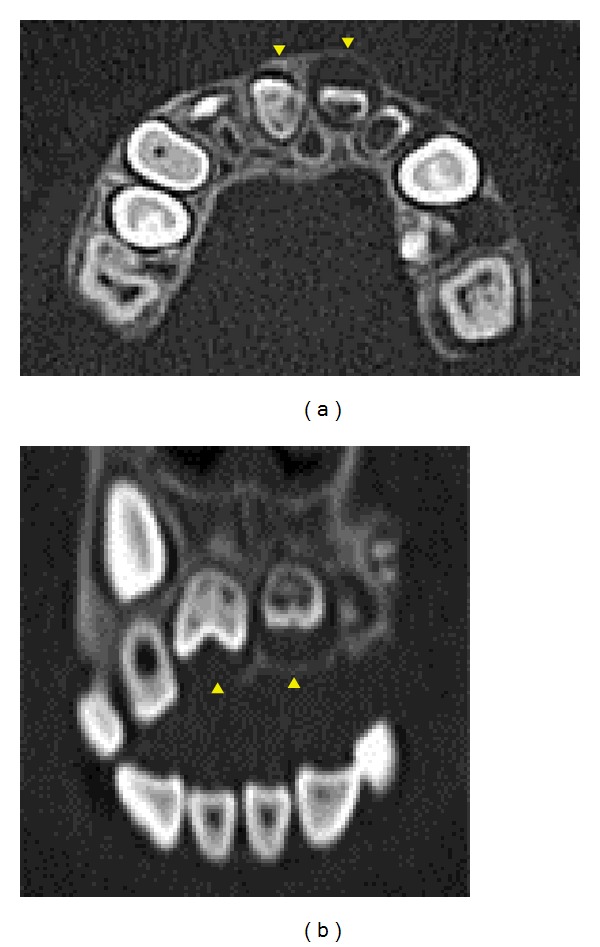
CT images of case 1. (a) Axial CT image shows labial expansion of dental follicles (arrowheads). (b) Multiplanar reconstruction image shows dental follicles clearly expanding toward alveolar ridge (arrowheads).

**Figure 8:**
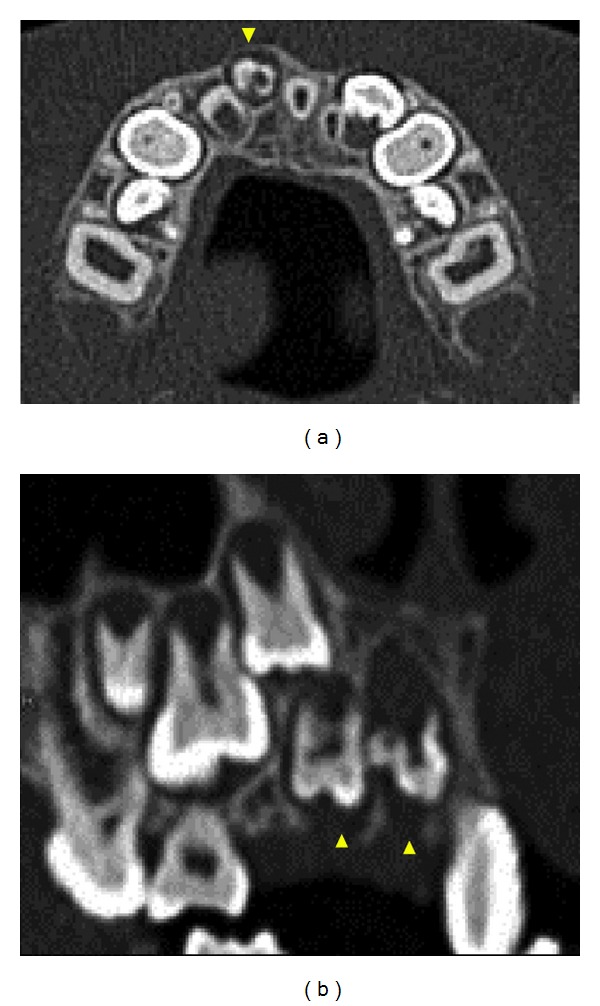
CT images of case 2. (a) Axial CT image shows labial expansion of dental follicles (arrowheads). (b) Multiplanar reconstruction image shows dental follicles clearly expanding toward alveolar ridge (arrowheads).

At ages 9 years and 5 months (case 1) and 9 years and 10 months (case 2), CT images were obtained to determine whether surgical treatment, such as fenestration, was indicated. To measure density, regions of interest were delimited using Image J software, Version 1.45. The images were analyzed and the profiles are shown in Figures 7 and 8.

Both patients had labial expansion of the dental follicles of affected permanent teeth, and the dental follicles had clearly expanded towards the alveolar ridge. Thinning of the cortical bone and labial bone expansion were also observed (Figures 7 and 8). The CT values of regions corresponding to enamel in affected teeth were 2300 to 3300 HU (case 1) and 2200 to 3100 HU (case 2). These values were lower than those for sound enamel in permanent teeth (approximately 4400 HU). The CT values of regions corresponding to dentin were similar for affected teeth and sound teeth (approximately 1800 HU). Because of the degree of calcification, we chose to follow up the patients without performing fenestration.

The CT values for the dental follicles of affected teeth in case 1 were 65 to 70 HU, which were similar to values for sound teeth (approximately 50 HU). In case 2, the CT values for the dental follicles of affected teeth were 110 to 120 HU, which were higher than the values for sound teeth. These findings suggest that dense fibrous connective tissue or hard tissue-like structures were present in the dental follicles.

## 3. Discussion

A small number of studies have used CT to investigate RO [7, 8]. Although one study reported detailed CT data for another condition, CT analysis of patients with RO has not been a research priority [10]. Plane radiographs cannot be used for pathologic three-dimensional evaluation. However, CT images allow clinicians to evaluate localization of dysplasia and hypocalcification, uneven distribution of calcification, radicular form, pulpal form, and periodontal tissues near affected teeth. Because CT data reflect the average radiodensity of all tissue within the volume elements, the density of a specific tissue type can be reliably determined [9]. We were able to obtain useful information on the degree of tooth calcification by comparing CT images of unerupted and sound teeth. This information could be very useful in diagnosing RO and deciding on a treatment strategy for unerupted teeth. In our two patients, the CT values for the enamel of affected teeth were lower than those for sound teeth, although CT values were similar for the dentin of affected and sound teeth. These findings indicate that hypocalcification is more severe in enamel than in dentin. Crawford and Aldred [3] found that demineralized sections had a residual enamel matrix, indicating the presence of both hypocalcification and hypoplasia. These histopathological states might be detectable in CT images.

In some patients, the unerupted tooth is surrounded by X-ray-permeable regions with clear boundaries, which resemble cysts or tumors [4–6]. Similarly, in our two patients, the dental follicles of the affected teeth were enlarged and resembled cysts. In our patients, however, the CT values for dental follicles were 65 to 70 HU (case 1) and 110 to 120 HU (case 2) in affected teeth. Thus, these structures were unlikely to be cysts, because the density was higher than that seen in cystic regions (about 20 HU) [9]. The CT value of muscle is about 50 HU, which suggests that the dental follicles in our patients might contain dense fibrous connective tissue or hard tissue-like structures resembling muscle.

There is no consensus regarding the treatment of RO [4, 11–16]. Some clinicians advocate removal of affected teeth [12, 16]. In contrast, Iizawa et al. [4] recommended that affected teeth, except for infected primary teeth, should be retained in the dental arch to promote ordinary jaw development. Nevertheless, most affected teeth are accompanied by severe inflammation, which necessitates extraction [11, 15]. When permanent teeth erupt in a patient with RO, it may be worthwhile to use noninvasive procedures, such as restoration with preformed crowns, to protect them from pulpal infection.

Impacted teeth may lead to infections of the tooth germ and sequelae due to the presence of retained epithelial tissues, such as formation of follicular cysts and odontogenic tumors. In our patients, the dental follicles of teeth affected by RO were enlarged and resembled cysts on radiographs. However, CT images revealed that they were fibrous connective tissue rather than cysts. Therefore, promotion of formation and calcification of dental germs in the jaws, rather than immediate fenestration, may be useful for treating RO and protecting hypocalcified teeth from pulpal infection after eruption.

## 4. Conclusion

Three-dimensional imaging and data from CT could greatly improve RO diagnosis and treatment. Because the CT values of regions corresponding to enamel in affected teeth were lower than those for sound enamel, promotion of formation and calcification of dental germs in the jaws, rather than immediate fenestration, may be useful for treating RO.
